# Total Intravenous Anaesthesia for Laparoscopic Cholecystectomy in a Patient With Congenital Long QT Syndrome: A Case Report

**DOI:** 10.7759/cureus.42707

**Published:** 2023-07-30

**Authors:** Xinyan Zhang, Chun Lei Tan

**Affiliations:** 1 Anaesthesiology, Changi General Hospital/Singhealth, Singapore, SGP

**Keywords:** perioperative medicine, metoclopramide, total intravenous anaesthesia, long qt syndrome, laparoscopic cholecystectomy

## Abstract

Long QT syndrome (LQTS) is characterised by QT interval prolongation and ventricular arrhythmia, leading to sudden cardiac death. Patients with acquired or congenital LQTS pose special challenges to anaesthetists perioperatively due to the risk of developing life-threatening arrhythmia. A variety of medications, including commonly used volatile anaesthetic agents are known to prolong QT interval and there has been growing evidence of using total intravenous anaesthesia (TIVA) instead of volatile agents for such patients.

This is a case report of a 30-year-old patient with congenital LQTS and subcutaneous implantable cardioverter defibrillator (SICD) in situ who underwent laparoscopic cholecystectomy and endoscopic retrograde cholangiopancreatography (ERCP) under TIVA safely within two months. There were no arrhythmic events observed perioperatively.

This case highlights the importance of comprehensive planning and meticulous preparation to avoid all possible QT-prolonging conditions during the perioperative period, especially in patients with acquired or congenital LQTS.

## Introduction

QT interval prolongation has been associated with ventricular arrhythmia and sudden cardiac death [1]. Prolonged heart rate corrected QT interval (QTc) is defined as >450 millisecond (ms) in males and >460 ms in females [2]. A prolongation of more than 20 ms from baseline increases the risk of torsades de pointes (TdP), while a QTc exceeding 500 ms is considered a major risk factor for TdP [3].

Patients with long QT syndrome (LQTS) pose special challenges to anaesthetists as the risk of developing arrhythmic complications is higher during the perioperative period. Acute illness, certain surgical procedures and anaesthetic agents might trigger life-threatening arrhythmia and result in unwanted outcomes. 

Here we present a case of a young patient with congenital LQTS who underwent both general anaesthesia and procedural sedation under total intravenous anaesthesia (TIVA) safely within two months. 

## Case presentation

Patient was a 30-year-old Chinese female (161cm, 75kg) diagnosed with congenital LQTS type 2 in 2016 after being investigated for recurrent syncope. Her QTc was prolonged in electrocardiogram (ECG) and her gene test was positive for KCNH2 mutation. Other than that, she had normal transthoracic echocardiography, no arrhythmia in 24-hour Holter and had negative treadmill exercise test at 10.9 metabolic equivalents of tasks (METs). She was started on bisoprolol 2.5mg every morning (OM) and subsequently had a subcutaneous implantable cardioverter defibrillator (S-ICD, Model 3501) implanted in 2020. 

Her past surgical history included open exploratory laparotomy, adhesiolysis and appendicectomy for congenital malrotation of gut in 2017. Patient's father had history of hypertension and what sounded like a sudden cardiac death in his 60s. None of patient's seven siblings had similar medical conditions. 

First procedure

**Figure 1:**
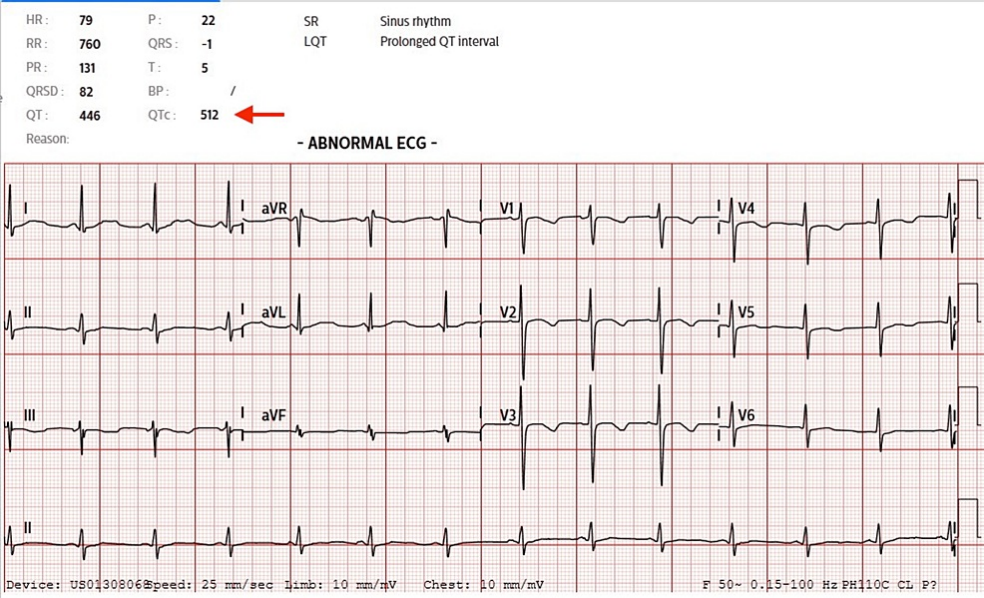
Preoperative Electrocardiogram

In August 2022, patient presented to emergency department (ED) with acute cholecystitis and was consented for laparoscopic cholecystectomy. Preoperative investigations were largely normal except elevated white blood cell count and prolonged QTc of 512ms (Figure 1). She was reviewed by cardiologist and kept on the same dose of bisoprolol. S-ICD was checked the day before operation. 

On the day of operation, patient was placed on American Society of Anaesthesiologists (ASA) standard monitoring (ECG, non-invasive blood pressure, pulse oximeter, bispectral index monitor). Magnet was placed over S-ICD to deactivate the defibrillation function after placement of the defibrillation pads. 

Patient was induced with propofol 170mg, fentanyl 100mcg, lidocaine 100mg and neuromuscular blockade was achieved with rocuronium 50mg. She was intubated with size 7.5 endotracheal tube uneventfully. Dexamethasone 8mg was given after induction as antiemetic. Anaesthesia was maintained with TIVA using target-controlled infusion (TCI) propofol (2.8-3ng/ml) and patient was ventilated with oxygen and air mixture. Operation lasted two hours and patient remained hemodynamically stable. Neuromuscular blockade was reversed with sugammadex 200mg and given oxycodone 8mg for analgesia. Total 80mg of esmolol was used during induction and emergence to control the heart rate and there were no arrhythmic events intraoperatively. Magnet was removed after extubation and S-ICD was checked by cardiology technician in post-anaesthesia care unit (PACU). Patient was transferred to high dependency ward for continuous ECG monitoring overnight and stepped down to general ward the next day. She remained well and stable throughout the hospital stay and was discharged home on postoperative day two.

Second procedure

In September 2022, patient was admitted again for abdominal pain and was diagnosed with acute cholangitis. Surgical team attempted endoscopic retrograde cholangiopancreatography (ERCP) but failed because patient was unable to tolerate the procedure. Percutaneous transhepatic biliary drainage (PTBD) was performed instead and patient received three doses of IV metoclopramide 10mg post-PTBD to treat nausea. Subsequently she felt S-ICD shocked once in the evening and telemetry showed polymorphic ventricular tachycardia (VT) initiated by R-on-T phenomenon at 20:37 lasting 12 seconds, followed by another 20 seconds of VT which was terminated with one appropriate shock. Patient remained hemodynamically stable and metoclopramide was deemed to be the trigger of polymorphic VT.

Two weeks later, she underwent ERCP successfully under sedation by anaesthesia team in endoscope center. S-ICD was disabled by cardiology technician after continuous ECG monitoring and defibrillation pads were placed. End-tidal CO2 was monitored via nasal prong catheter besides standard ASA monitoring. Patient was placed in prone position and sedated with TCI propofol 1-2ng/ml titrated to effect. Intermittent fentanyl boluses were given as analgesics, 200mcg in total. Patient remained stable during the procedure and S-ICD was reprogrammed in PACU. She was discharged home two days after the procedure.

## Discussion

Patients with LQTS pose special challenges to anaesthetists during perioperative period due to increased risk of developing malignant ventricular arrhythmia. Both congenital and acquired LQTS can affect perioperative management and they are not mutually exclusive. In fact, the first manifestation of congenital LQTS may occur in the setting of acquired QT prolongation [4,5]. Our patient had known congenital LQTS2 and she developed polymorphic VT during the second admission, likely due to repeated dose of metoclopramide, which was an acquired trigger. 

We present a review of both congenital and acquired LQTS, as well as the relevant anaesthetic management perioperatively. 

Acquired LQTS 

Acquired LQTS is more commonly seen in clinical practice. Any factor that results in a net reduction in the outward current will prolong repolarization and, therefore, lengthen the QT interval [5]. The prevalence of acquired LQTS was reported to be 7.95% at ED admission and female patients on antibiotics or antipsychotics were at particularly high risk [6]. A range of medications can cause QT prolongation and the most commonly implicated drugs are Class 1A/III antiarrhythmics, antipsychotics, promotility agents, and certain antimicrobials. Other non-drug related causes include electrolyte disturbances (hypokalaemia, hypomagnesaemia, and hypocalcaemia), ischaemic or structural heart disease, and intracranial events (subarachnoid haemorrhage, stroke, and brain surgery) [4].

The treatment for acquired LQTS is to reverse the known or possible triggers and avoid those in the future. When patient develops ventricular arrhythmia or TdP, the management should follow advanced cardiovascular life-support (ACLS) guidelines, with synchronised cardioversion and magnesium sulphate administration to shorten the QT interval and stabilise the cardiac cell membranes. 

Congenital LQTS

Congenital LQTS is an inherited channelopathy that represents a leading cause of sudden cardiac death. It is typically characterised by QT interval prolongation and ventricular tachyarrhythmia, leading to syncope and even cardiac arrest. Symptomatic patients left without treatment have a high mortality of 21% within one year from the first syncope [1]. However, with proper treatment, mortality can be as low as 1% [7]. In a prospective study conducted in Italy, disease-causing mutation was identified in 43% and 29% of the infants with a QTc exceeding 470ms and 460ms, respectively, demonstrating a prevalence of at least 1:2534 in healthy live births [8].

Pathophysiology

The QT interval represents the depolarization and the repolarization phases of cardiac action potential. Decreases in repolarizing outward K+ currents or increases in depolarizing inward sodium or calcium currents can lead to QT interval prolongation, thus representing a pathophysiological substrate for LQTS. Variations in genes encoding for ion channels and their modulatory subunits or proteins regulating the function of ion channels have been identified as disease-causing mutations in up to 75% of LQTS cases. The majority of LQTS are autosomal dominant, and by far, KCNQ1 (LQT1), KCNH2 (LQT2), and SCN5A (LQT3) are the most common LQTS genes, accounting for 90% of all genotype-positive cases [9]. Our patient has KCNH2 mutation, the second most common gene mutation in LQTS that encodes the α-subunit of the K+ channel conducting the IK rectifier (IKr) current. Mutations in KCNH2 cause a reduction in Ikr current, hence prolongation of QT interval [7].

Clinical presentation and diagnosis

Symptoms in LQTS are caused by runs of ventricular arrhythmias, to be specific, TdP. It is usually self-limiting and produces transient syncope but can also develop into ventricular fibrillation (VF) and cause cardiac arrest. The conditions associated with arrhythmic events can be gene-specific. For example, TdP is typically triggered by exercise in LQTS1, by sudden loud noises or being startled in LQTS2, and at rest or during sleep in LQT3 patients [9].

The diagnosis of congenital LQTS relies on the measurement of QTc, clinical symptoms, pathogenic gene mutation and high LQTS risk score. Secondary causes of QTc prolongation must be excluded. The LQTS risk score (Schwartz score) was developed to predict LQTS probability and patients with high Schwartz score can be selected for genetic testing [10].

Management

Lifestyle changes are recommended in all patients diagnosed with LQTS: avoidance of strenuous exercise in LQTS1 group, reduction in exposure to abrupt loud noises in LQTS2 group, avoidance of QT-prolonging drugs (www.qtdrugs.org), and correction of electrolyte abnormalities [10]. β-blockers are the first line pharmacological therapy in symptomatic patients or patients with a genetic diagnosis but normal QTc. For patients with intolerance to β-blockers or who have breakthrough events despite optimal drug dose, left cardiac sympathetic denervation (LCSD) should be considered [8]. LSCD has been shown to result in a 91% reduction in cardiac events in high-risk LQTS patients [9].

Implantable cardioverter defibrillator (ICD) implantation is usually the final level of therapy. There is an overall consensus for ICD implantation in cases with a documented cardiac arrest, especially in patients with LQTS-related syncope who have already been treated with β-blocker [8]. ICD therapy has lifetime implications with possible complications like lead fracture and inappropriate shock, therefore the risks and benefits must be carefully considered before initiating this therapy in young patients [9,10]. 

Our patient received S-ICD which is specifically designed to overcome some of the complications related to the traditional transvenous ICD (TV-ICD) such as lead complications and systemic infections as the lead in S-ICD was implanted subcutaneously. Currently, the evidence on the comparison of S-ICD versus TV-ICD is limited. One of the recently published meta-analyses has looked into 13 studies counting 9073 patients and found that S-ICD had a lower risk of lead complications and major procedural complications, but a higher risk of pocket complications compared with TV-ICD. Otherwise S-ICD was at least as effective as TV-ICD for the prevention of sudden cardiac death without the need of pacing [11].

The understanding of the genotype-phenotype correlation in LQTS has made gene-specific management possible. For example, LQT2 patients are exquisitely sensitive to serum K levels and hypokalaemia should be promptly corrected [9]. Our patient indeed had ICD shock after a few days of diarrhoea, with a 3.3mmol/L serum potassium.

Anaesthesia considerations

Approximately 40 fatal cases of preoperative TdP have been reported in the past 30 years, but the actual incidence of TdP might be higher as TdP is usually self-limiting [4]. Currently there are no consensus guidelines on the anaesthetic management of patients with acquired or congenital LQTS and most of the recommendations are from case reports. Anaesthetists should be aware of the disease and the relevant treatment options. The goal of perioperative care is to minimise the risk of malignant arrhythmias and prevent sudden cardiac death. 

Preoperative assessment

A comprehensive assessment including history, physical examination and investigations focusing on the cardiovascular system is the cornerstone of perioperative care. Any new arrhythmia-related symptoms should be fully investigated and the medication list should be screened for potential QT prolongation effect. Electrophysiology consultation should be obtained for patients with newly diagnosed QT prolongation. For patients already on treatment, β-blockers must be continued perioperatively, and ICD needs to be checked before operation. Baseline ECG and laboratory tests are required for all patients. Any electrolyte abnormalities must be corrected to minimise the risk of arrhythmia, especially hypokalaemia, hypomagnesaemia and hypocalcaemia. 

Multi-disciplinary discussion including the surgeon, cardiologist and anaesthetist should be conducted prior to the operation. In this case, detailed surgical plan, preoperative investigation and postoperative disposition were discussed among the specialists and surgeons agreed to limit the pneumoperitoneum pressure and place patient in high-dependency ward postoperatively. 

Induction

A quiet and warm operation theatre (OT) with minimal personnel is preferred, especially for LQTS2 patients like ours because auditory stimuli may trigger arrhythmia. Premedication with midazolam could be considered for those who are anxious. Besides standard ASA monitoring, core temperature should be monitored to avoid hyper- or hypothermia as both have been shown to prolong QT interval [4]. For patients with ICD in situ, the anti-arrhythmic function needs to be deactivated after placement of continuous ECG monitoring and defibrillation pads. Defibrillation machine should be readily available in the OT before induction. 

Inductions drugs like propofol, thiopental and etomidate have been used without problems despite their potential QT prolongation effects, but propofol might be superior to thiopental and etomidate in terms of the effects on QTc [3]. Although human studies are lacking, ketamine might not be a good option due to its sympathomimetic properties. 

Most non-depolarizing neuromuscular blockers have not been associated with QTc prolongation and can be used safely [3]. Suxamethonium, however, has been found to significantly prolong QTc and should be avoided. Its QTc prolongation effect has been attributed to sympathoadrenal activation and might be prevented by β-blockers and opioids [4]. If rapid sequence induction is indicated, rocuronium can be used as a safer alternative. 

The sympathetic stimulation caused by laryngoscopy and tracheal intubation may trigger arrhythmia and should be minimised as much as possible. Co-induction with fentanyl 2mcg/kg has been shown to significantly attenuate the QTc prolongation associated with laryngoscopy [3]. Intravenous lignocaine, esmolol and remifentanil have been used in our institution to blunt hemodynamic changes for smooth intubation and extubation. 

Maintenance 

All halogenated volatile anaesthetics prolong QTc in different degrees [3], and sevoflurane (the main volatile agent used in our institution) has a concentration-dependent QT prolongation effect within clinical relevant concentration [3,12]. Nitrous oxide has been shown to prolong QTc in 80% of patients undergoing non-cardiac surgery [13]. Therefore, TIVA with propofol and remifentanil was chosen for this case. Both remifentanil and propofol have been reported to reverse the QTc prolongation caused by sevoflurane, although the effect of propofol infusion on QTc is controversial in paediatric population [3,14]. 

Optimal analgesia is essential to blunt the hemodynamic surge from surgical stimulation, and multimodal analgesia should be considered. Simple analgesics (paracetamol, nonsteroidal anti-inflammatory drugs [NSAIDs]) and opioids commonly used in our institution (remifentanil, fentanyl, morphine, oxycodone) have been safely used at clinical doses, but QTc prolongation was observed in cardiac patients who received 5mcg/kg of fentanyl [3].

Peripheral nerve block with bupivacaine or ropivacaine helps with postoperative pain control and can be considered for upper and lower extremity procedures. Central neuraxial anaesthesia has been used safely in patients with known LQTS, including obstetric population [15]. Our patient would benefit from epidural anaesthesia if her operation was converted to open laparotomy as low-dose epidural provides excellent analgesia without too many hemodynamic swings. 

Laparoscopy surgery 

Intraperitoneal CO2 insufflation during laparoscopic surgery has been shown to cause a reversible increase of QTc which starts after 30 minutes and peaks at 120 to 150 minutes [16]. Most of the studies did not report arrhythmia during pneumoperitoneum inflation, but a case of intraoperative VF was reported in a young patient during laparoscopic hysterectomy. This patient had normal preoperative QTc and was subsequently diagnosed with congenital LQTS type 8 [17]. Therefore, extra care must be taken during pneumoperitoneum period and intra-abdominal pressure should be monitored and kept as low as possible. In our case, the intraperitoneal pressure was limited to 10-12mmHg and operation was performed by an experienced surgeon to minimise the hemodynamic effect of pneumoperitoneum. 

Emergence and postoperative care 

Anticholinergics and anticholinesterase-anticholinergic combination should be used with caution to reverse neuromuscular blockage as both atropine and glycopyrrolate have been reported to prolong QTc [18]. In this case, we used sugammadex to reverse rocuronium. Previous RCT has shown that both therapeutic (4mg/kg) and supra-therapeutic (32mg/kg) sugammadex were not associated with clinically important QTc prolongation [19]. 

Prophylactic antiemetics should be administered to prevent postoperative nausea and vomiting (PONV). In this case, we used TIVA and gave dexamethasone 8mg at the beginning of operation as antiemetics. Our patient did not have PONV, but for patients suffering from PONV, antiemetics should be given at a low dose with close monitoring. 5-HT3 receptor antagonists are known to increase QTc and the QT prolongation effect is dose-dependent. The latest recommendation from the US Food and Drug Administration states that the single intravenous dose of ondansetron should not exceed 16mg [20]. Metoclopramide is an alternative to 5-HT3 receptor antagonist, but our patient developed ventricular arrhythmia after three doses of metoclopramide during her second admission. It is not listed as a QT-prolonging medication but it has been mechanistically shown to change QT dynamics and may prolong QT and increase the risk of TdP [21].

Care must be taken to avoid hemodynamic swings during extubation, and similar strategies can be applied as per intubation paragraph. Patient should be kept in a quiet corner cubicle in PACU with continuous ECG monitoring until ICD has been checked and re-activated. 

## Conclusions

Here we reported a case of patient with congenital LQTS who underwent laparoscopic surgery uneventfully under TIVA. Although there are no consensus guidelines on the optimal perioperative management, the summary and recommendations in this case report might be useful for clinicians managing this group of patients. Multidisciplinary collaboration with cardiologist and surgeon as well as meticulous planning are the key to patient safety. 
